# NBI and NBI Combined with Magnifying Colonoscopy

**DOI:** 10.1155/2012/173269

**Published:** 2012-12-09

**Authors:** Mineo Iwatate, Taro Ikumoto, Santa Hattori, Wataru Sano, Yasushi Sano, Takahiro Fujimori

**Affiliations:** ^1^Gastrointestinal Center, Sano Hospital, Kobe 655-0031, Japan; ^2^Department of Surgical and Molecular Pathology, Dokkyo University School of Medicine, Tochigi, Japan

## Abstract

Although magnifying chromoendoscopy had been a reliable diagnostic tool, narrow-band imaging (NBI) has been developed in Japan since 1999 and has now replaced the major role of chromoendoscopy because of its convenience and simplicity. In this paper, we principally describe the efficacy of magnifying chromoendoscopy and magnifying colonoscopy with NBI for detection, histological prediction, estimation of the depth of early colorectal cancer, and future prospects. Although some meta-analyses have concluded that NBI is not superior to white light imaging for detection of adenomatous polyps in screening colonoscopy, NBI with magnification colonoscopy is useful for histological prediction, or for estimating the depth of invasion. To standardize these diagnostic strategies, we will focus on the NBI International Colorectal Endoscopic (NICE) classification proposed for use by endoscopists with or without a magnifying endoscope. However, more prospective research is needed to prove that this classification can be applied with satisfactory availability, feasibility, and reliability. In the future, NBI might contribute to the evaluation of real-time histological prediction during colonoscopy, which has substantial benefits for both reducing the risk of polypectomy and saving the cost of histological evaluation by resecting and discarding diminutive adenomatous polyps (resect and discard strategy).

## 1. Introduction

Narrow-band imaging (NBI) is a technique by which spectral features are modified by narrowing the band width of spectral transmittance using filters adjusted to the characteristics of hemoglobin absorption [1–3]. Diagnosis based on angiogensis or vascular morphological change can be ideal for early detection and diagnosis of neoplastic lesions, as angiogenesis plays a critical role in the transition of premalignant lesions in a hyperproliferative state to a malignant phenotype [4–6]. By simply operating a button on the control panel of the endoscope, NBI yields a unique image that emphasizes the pattern of capillaries (vessels), as well as the surface of mucosal tissues. Because of its similarity to chromoendoscopy, NBI can be referred to as “optical/digital chromoendoscopy.” The utility of NBI is enhanced when it is employed with a magnifying endoscope providing low- to high-power magnification (×80–100 maximum) utilizing a one-touch electrical power system [7].

This paper highlights the efficacy of magnifying chromoendoscopy and NBI colonoscopy with/without optical zoom magnification for diagnosis of colorectal lesions and discusses future perspectives.

## 2. Magnifying Chromoendoscopy

### 2.1. When and How to Use Magnifying Chromoendoscopy

Colorectal lesions are initially diagnosed by conventional-view colonoscopy, and then, if possible, by magnifying view and/or chromoendoscopy using indigo carmine. We routinely use a magnifying colonoscope because the insertion technique and manipulation are similar to those for an ordinary colonoscope [8]. In a prospective study, Konishi and colleagues reported that insertion of a magnifying colonoscope into the cecum was achieved successfully in 97% of cases, and that there were no significant differences in the average time taken to reach the cecum or the average total procedure time [9]. It might be argued that magnifying endoscopy is difficult to learn, and in fact Togashi et al. have reported that examination of 200 polyps was necessary in order to achieve a sensitivity rate of 90% for neoplastic lesions [10]. In 2012, Olympus Corp. produced a new dual-focus endoscope capable of providing low- and medium-power optical magnification views (×45 maximum) automatically. It is anticipated that this endoscope will considerably shorten the learning period for magnifying endoscopy.

**Figure 1:**
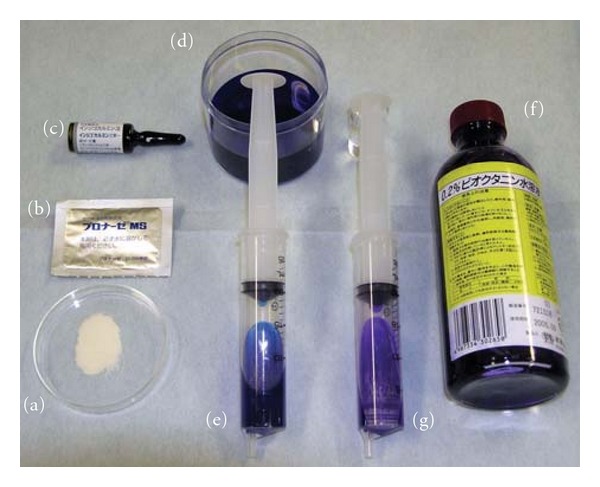
Preparations for magnifying observation. (a, b) Pronase MS. Washing of the target lesion surface can be done with 500 cc of lukewarm water containing a packet of Pronase MS (20000 U). (c) Indigo carmine (Daiichi Pharmaceutical Corp., Tokyo, Japan). (d) The dye is a blue stain that accentuates the contours of a lesion, providing a detailed view of its border and shape. The dye is used as a 0.1–0.4% aqueous solution. (e) This solution is flushed through the biopsy channel of the scope using a 20-cc syringe. Generally, 3–5 cc is delivered in 5 s along with 15 cc of air. (f) Crystal violet (Honzo Pharmaceutical Corp., Nagoya, Japan). The dye is a vital stain and is preferentially taken up by the Lieberkuhn gland openings (crypts), which appear as dots or pits. (g) A few small drops of crystal violet in 0.05% solution are applied using a nontraumatic catheter (Olympus 6233064; Olympus Optical Co., Ltd., Tokyo, Japan).

**Figure 2:**
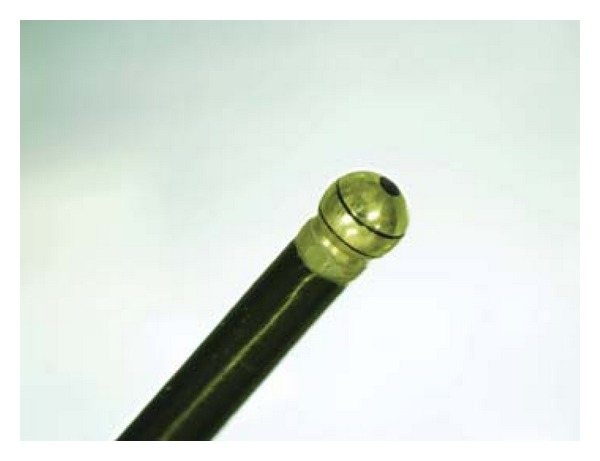
Nontraumatic, globular-tip catheter. This catheter is used to remove mucus and to drop crystal violet solution onto the lesion. Better positioning for magnifying observation can be obtained by pushing and holding the surrounding mucosa.

When a colonoscopist intends to perform chromoendoscopy, 3–5 mL of an aqueous solution of dye is sprayed onto the mucosa via the biopsy channel, along with 15 mL of air, using a 20-cc syringe. Common dyes used for characterization of the colorectum are indigo carmine as a contrast stain (0.1–0.4%), and crystal violet (0.05%) and methylene blue (0.1%) as absorptive stains. Although indigo carmine and methylene blue are often used to screen for sporadic adenoma, crystal violet, as an absorptive stain, offers advantages for patients with early invasive cancer or for detailed observation using a nontraumatic catheter after washing the lesion with lukewarm water containing pronase (Pronase MS) (Figures 1 and 2) [11].

**Figure 3:**
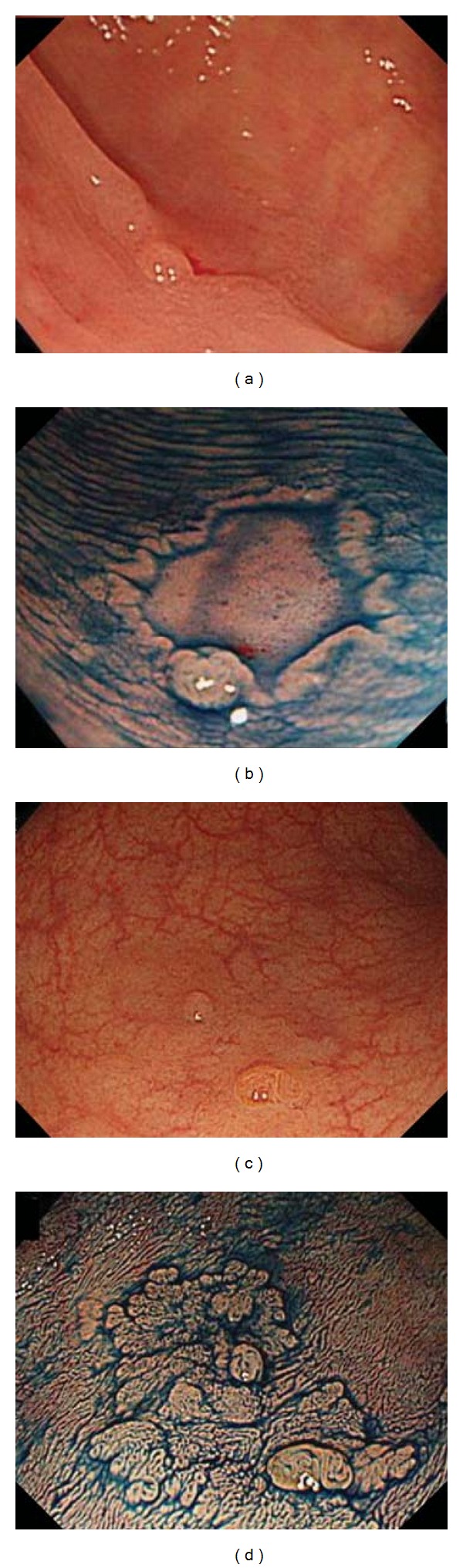
Usefulness of indigo carmine. (a) Disruption of the mucosal fold and a slightly reddish area are observed, but the whole lesion is unclear. (b) After spraying with indigo carmine dye, a 7 mm depressed lesion (0–II c) is identified clearly. (c) A slightly elevated lesion with an obscure superficial vascular component is evident, but the whole lesion is not recognized. (d) A slightly elevated lesion measuring 18 mm is obviously detected using indigo carmine.

Is it advisable to spray dye over the whole of the colon and rectum to identify significant lesions? When should magnification be employed? Certainly, pan-mucosal chromoendoscopy significantly increases the rate of detection of small neoplastic and flat lesions, but this technique requires an excessive volume of dye and a significantly prolonged procedure [12–16]. Therefore, colonoscopists use “selective” chromoendoscopy only for further examination of any subtle mucosal irregularity detected during standard colonoscopy. After a mucosal abnormality has been detected, target chromoendoscopy with magnification is indispensable for confirming the surface structure, perimeter shape, and mucosal crypt (pit) pattern of the lesion in detail (Figure 3). 

### 2.2. Classification and Clinical Usefulness of Magnifying Chromoendoscopy

**Figure 4:**
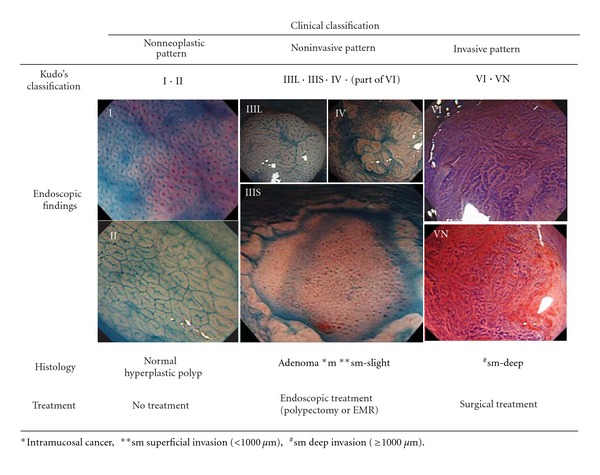
Pit pattern classification and invasive pattern.

**Figure 5:**
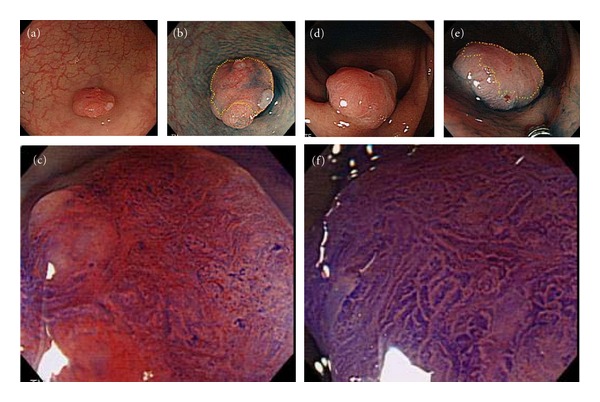
Cases of invasive pattern. (a) Endoscopic examination demonstrates a small (7 mm) flat elevated lesion in the sigmoid colon. (b) Chromoscopy with indigo carmine demonstrates a definite central depression. (c) Magnification with crystal violet staining demonstrates an invasive pattern in a demarcated area. Based on these findings, the tumor was diagnosed as an early colon cancer with deep submucosal invasion, and surgical resection was recommended. Histopathological examination of the resected specimen demonstrated well differentiated adenocarcinoma, invasive to the submucosa (sm deep; 4000 *μ*m). (d) A sessile lesion Is(+IIc), 15 mm in diameter, identified in the upper rectum. (e) Chromoscopy with indigo carmine: reddish change and slight depression are observed on the surface of the tumor. (f) Magnification with crystal violet staining demonstrates an invasive pattern. Histopathological examination of the resected specimen demonstrated well differentiated adenocarcinoma (sm deep; 4500 *μ*m).

Kudo has proposed a gross classification of pit patterns into 7 types. It has been suggested that type I and II pit patterns are characteristic of nonneoplastic lesions such as normal mucosa or hyperplastic polyps. However, most lesions showing pattern types IIIS, IIIL, IV, and a subset of VI are intramucosal neoplastic lesions such as adenoma or intramucosal carcinoma. Lesions with a type VN pattern and a subset of type VI suggest deep invasive carcinoma (Figure 4) [7]. Because pit pattern classification is complicated for nonskilled endoscopists, Fujii et al. have modified it to a simpler form for clinical use. This clinical classification of pit patterns includes 3 types (nonneoplastic, noninvasive, and invasive) for which corresponding treatments are required (no treatment, endoscopic resection, and surgery, resp.) (Figure 5) [11, 17]. The precise definitions of these clinical pit patterns are as follows.

1. *Invasive pattern*: irregular and distorted crypts in a demarcated area, as is evident in Kudo's type VN and selected cases of VI (e.g., deep submucosal invasive cancer).
2. *Noninvasive pattern*: regular crypts with or without a demarcated area, or irregular pits without a demarcated area, as is evident in Kudo's type IIIS, IIIL, or IV, or selected cases of VI (e.g., adenomatous polyps and superficial submucosal invasive cancers).
3. *Nonneoplastic pattern*: normal mucosa and star-shaped crypts, as is evident in Kudo's type I or II, respectively (e.g., hyperplastic, juvenile, and inflammatory polyps).

We have conducted a prospective study to examine whether magnification and/or indigo carmine dye-spraying is more reliable than the conventional view for distinguishing nonneoplastic from neoplastic lesions of the colon and rectum [18]. The overall diagnostic accuracy of magnification in addition to chromoendoscopy using indigo carmine was 95.6%, being 10% and 5% more reliable than conventional endoscopy and chromoendoscopy, respectively. In addition, this method was significantly superior to conventional endoscopy and chromoendoscopy (*P* < 0.0001 and *P* = 0.0152). 

Also, our recent large prospective series has demonstrated that the clinical classification of pit patterns (as invasive or noninvasive) is effective for differentiating intramucosal or sm superficial invasion (<1000 *μ*m) from sm deep invasion (≥1000 *μ*m). In that study, histopathology confirmed epithelial neoplasia in 99.4% of 4037 lesions with a noninvasive pattern, and confirmed deep sm invasion in 86.5% of 178 lesions with an invasive pattern [19]. Furthermore, the calculated sensitivity, specificity, positive predictive value, negative predictive value, and accuracy were 85.6%, 99.4%, 86.5%, 99.4%, and 98.8%, respectively.

Therefore, based on these results, we are able to conclude that at present, a combination of magnifying colonoscopy and chromoendoscopy is the one of the most reliable methods for distinguishing nonneoplastic from neoplastic lesions, as well as for estimating the depth of early colorectal cancer.

## 3. Narrow-Band Imaging (NBI)

### 3.1. Detection

The colon and rectum are sites where neoplastic lesions occur most frequently. The National Polyp Study Group in the USA has reported that resection of all neoplastic polyps led to a 76–90% reduction in the incidence of colorectal cancer and a subsequent 53% reduction in mortality [22, 23]. Therefore, for prevention of colorectal cancer, it is important to find and resect neoplastic lesions as accurately as possible.

Neoplastic lesions are recognizable as a brownish area using NBI without magnification, which emphasizes neoplastic angiogenesis. Is NBI without magnification more useful for detecting neoplastic lesion than white light imaging (WLI)? Uraoka et al. have reported that NBI is superior to WLI (*P* = 0.046) for detection of flat and diminutive lesions [24]. However, Rex and Helbig have reported that there is no significant difference in the detection rate between NBI and WLI (65% versus 67%  *P* = 0.61) [25]. Previous studies of the adenoma detection rate (ADR) have yielded conflicting results. Recently, to evaluate the ADR, Ikematsu et al. conducted a multicenter prospective trial in which 813 patients were randomized to a primary NBI group (NBI-WLI) and a primary WLI group (WLI-NBI) in the right-sided colon using the tandem method [26]. They found that the ADR for primary NBI and WLI was 42.3% and 42.5%, respectively, and the difference was not significant. Furthermore, three recent meta-analyses have concluded that NBI has no superiority to WLI in terms of ADR [27–29]. Therefore it can be concluded that the ADR for NBI is equivalent to that for WLI, and that both can be used equally effectively for screening colonoscopy. 

### 3.2. Histological Prediction

**Figure 6:**
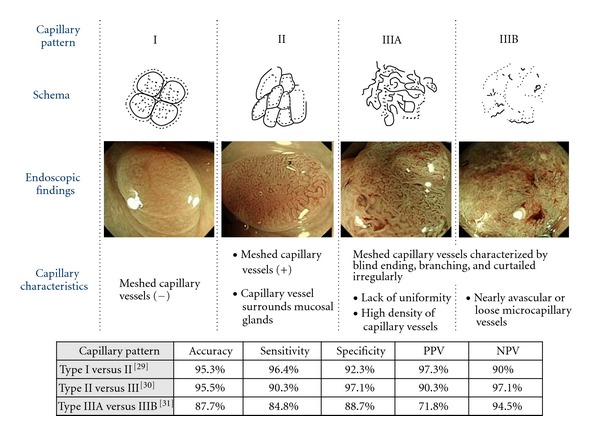
Capillary pattern (CP) classification and diagnostic ability (published data).

In accordance with our previous investigations, the microvascular architecture (capillary pattern: CP) was classified into three types (CP types I, II, and III), and CP type III lesions were further classified into two groups: types IIIA and IIIB (Figure 6) [30–33]. Our observations demonstrated that assessment of CP by magnifying NBI is useful for differentiating small colorectal nonneoplastic from neoplastic polyps (accuracy 95.3%, sensitivity 96.4%, specificity 92.3%) and is highly accurate for distinguishing low-grade dysplasia from high-grade dysplasia/invasive cancer (accuracy 95.5%, sensitivity 90.3%, specificity 97.1%), and can thus be used to predict the histopathology of colorectal neoplasia [31, 32]. Because magnifying colonoscopy with NBI is convenient to use and as accurate as magnifying colonoscopy, we principally use only magnifying colonoscopy with NBI, and not chromoendoscopy, to distinguish neoplastic from nonneoplastic polyps during routine colonoscopy [34]. 

### 3.3. Estimation of the Depth of Early Colorectal Cancer

In Japan, there is growing evidence to support the theory that lesions with submucosal invasion limited to <1000 *μ*m without lymphovascular involvement and a poorly differentiated component lack LN metastases [35–38] and can be cured by endoscopic resection alone. The Paris endoscopic classification of superficial neoplastic lesions has also determined 1000 *μ*m to be the cutoff limit between sm1 and sm2 [39]. It is important to determine the vertical depth of invasion of submucosal colorectal cancers prior to endoscopic resection, because endoscopic resection of early colorectal cancer with massive submucosal invasion carries a high risk of bleeding and perforation.

Ikematsu and colleagues conducted a prospective study to determine whether CP type IIIA/IIIB identified by magnifying NBI was effective for estimating the depth of invasion in 130 early colorectal neoplasms [33]. These included 15 adenomas, 66 intramucosal cancers (pM), and 49 submucosal cancers (pSM): 16 pSM superficial (pSM1) and 33 pSM deep (pSM2-3) cancers. The sensitivity, specificity, and diagnostic accuracy of CP type III for differentiating pM-ca or pSM1 (<1000 *μ*m) from pSM2-3 (≥1000 *μ*m) were 84.8%, 88.7%, and 87.7%, respectively. The accuracy of CP type IIIA (NPV) was 94.5% (86/91), and that for lesions of CP type IIIB (PPV) was 71.8% (29/39). In their study, the rate of diagnostic agreement among the three observers was good, without significant variability (interobserver variability: *κ* = 0.68, 0.67, 0.72; intraobserver agreement: *κ* = 0.79, 0.76, 0.75).

Submucosal saline injection is another useful method for estimating the depth of tumor invasion, not only when used for endoscopic mucosal resection (EMR) but also as a simple diagnostic tool for deeply invasive cancers. In Japan, Uno and Munakata were the first to propose the “nonlifting sign” in 1994, and considered it to be positive in cases where the surrounding mucosa, but not the lesion, was elevated [40]. Although adenoma and intramucosal cancer are easily lifted by submucosal injection of saline, deeply invasive cancer is not lifted because of the presence of a desmoplastic reaction and the invasive nature of the lesion (Table 1).

**Figure 7:**
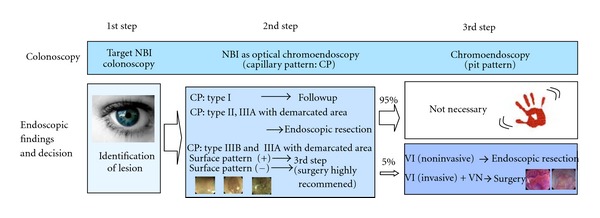
Modified 3-step strategy of NBI colonoscopy.

Considering the available evidence, we have suggested a three-step strategy for management of colorectal lesions using conventional colonoscopy, NBI colonoscopy, and chromoendoscopy (Figure 7). Chromoendoscopy is necessary in cases where deep invasion of the lesion into the submucosal layer is suspected, accounting for only 5% of all neoplastic lesions. 

### 3.4. International Collaboration on NBI Observation of Colorectal Tumors: NBI International Colorectal Endoscopic (NICE) Classification

**Figure 8:**
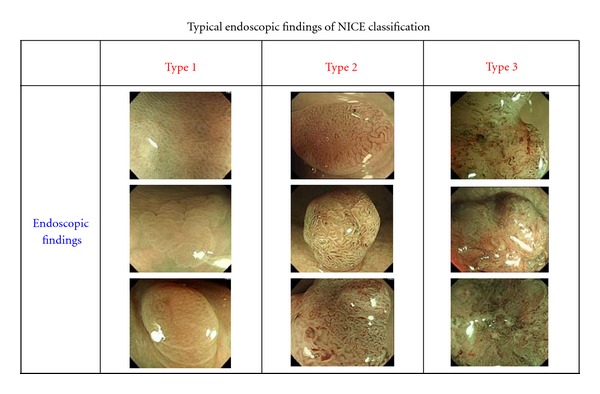
Figures to illustrate the NBI International Colorectal Endoscopic (NICE) classification.

In 2011, we proposed an international classification system for conventional endoscopic observation assisted by NBI, and for applying this system to NBI magnifying observation (Table 2, Figure 8) [41]. When closely observing a colorectal lesion with the latest high-resolution electronic endoscope, the pit-like pattern on the surface is visible without the use of magnifying endoscopy. The use of NBI allows enhanced structural visualization, and hence the microvessels on the tumor surface can be observed in addition to the pit-like pattern. In Western countries, the magnifying endoscope is not widely used in clinical practice [42]. Even in Japan, use of the magnifying colonoscope in daily practice is still insufficiently widespread. Against this background, we have devised a simple system for categorical classification of colorectal tumors on the basis of NBI observation either with or without use of a magnifying endoscope (NBI International Colorectal Endoscopic [NICE] classification). We, along with endoscopists from Western countries, have reviewed the NICE classification system, and in 2012 some of our combined results were published in *Gastroenterology*, [43]. The NICE classification system is a simple categorical classification consisting of types 1–3 and based on three characteristics: (i) lesion color; (ii) microvascular architecture; and (iii) surface pattern. Type 1 is considered an index for hyperplastic lesions, type 2 an index for adenoma or mucosal/SM scanty invasive carcinoma, and type 3 an index for deeply SM-invasive carcinoma. To objectively verify the clinical usefulness of the NICE classification, an international collaborative effort has been launched by the Colon Tumor NBI Interest Group (CTNIG), whose members include Yasushi Sano (Japan), Shinji Tanaka (Japan), Douglas K. Rex (USA), Roy M. Soetikno (USA), Thierry Ponchon (France), and Brian P. Saunders (UK). The key advantage of the NICE classification is that it can be used by endoscopists without a magnifying endoscope. Some validation studies of the NICE classification have already been performed. Hewett et al. have reported that nonneoplastic and neoplastic lesions (NICE1 and NICE2) can be diagnostically differentiated in real time with high confidence by skilled endoscopists, achieving an accuracy of 89%, a sensitivity of 98%, and a negative predictive value of 95% [41]. In a study employing an endoscopic image library assessed by medical students, Nakayama et al. have confirmed that the NICE classification is valid for prediction of deep submucosal invasive carcinoma (NICE2 and NICE3). Diagnoses after teaching achieved an overall accuracy of 84.3% and an accuracy of 90.0%, high confidence being achieved for half of all cases examined [42].

Table 3 shows the relationship between the NICE classification and major NBI magnifying classifications used in Japan (Uraoka et al. [44, 45]/Hirata et al. [46]/Wada et al. [47] classification). As can be seen, the NICE classification has potential for use as a basic categorical classification of lesions demonstrated by NBI magnifying endoscopy. As a next step, we consider that it would be valuable to subclassify NICE type 2 lesions on the basis of features revealed by magnifying endoscopy to allow more detailed characterization of colorectal intramucosal tumors and scantily invasive carcinomas.

### 3.5. Resect and Discard Strategy

**Figure 9:**
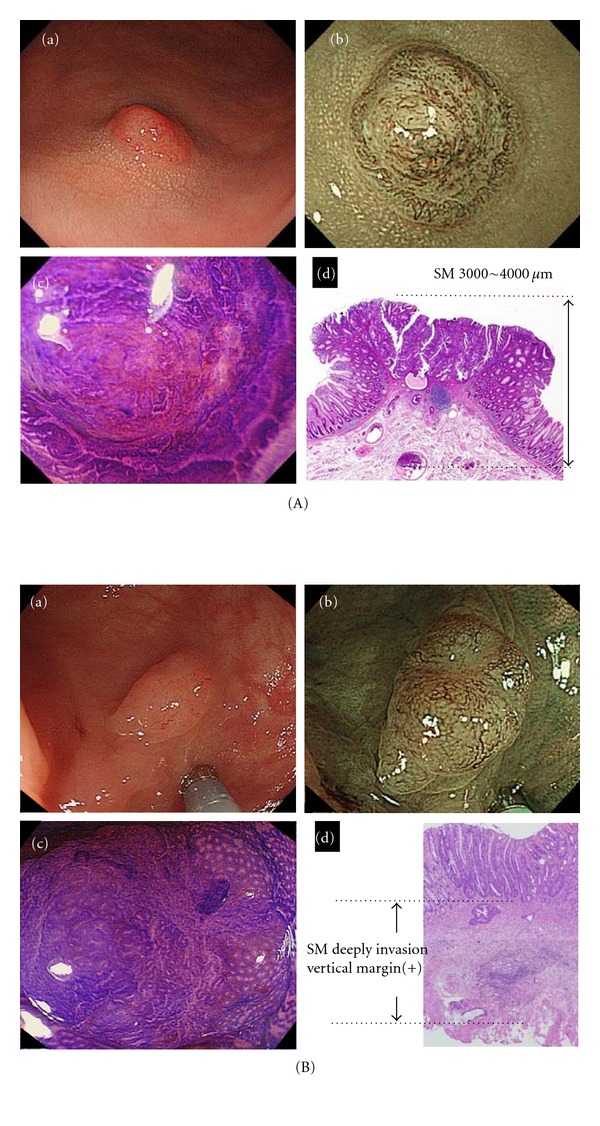
Two typical cases of small invasive colorectal cancer (≤5 mm). (A): (a) Conventional view: There is a small polyp (lesion size: 4 mm) located in the sigmoid colon. It is rather difficult to visualize any depressed area in this lesion. (b) NBI view: Magnification with NBI clearly demarcates the margin of the depressed area. The vascular pattern is capillary pattern Type III_B_ of the Sano classification, indicative of deep invasion into the submucosa. The center of the depressed area has a dome-like appearance, and the macroscopic type is “0-I s + II c”, which requires attention in view of its frequent deep invasion into the submucosa. (c) Crystal violet view: magnification with crystal violet staining also demonstrates an invasive pattern and V_N_ pits, strongly indicative of deep submucosal invasion. We decided to treat this lesion surgically without endoscopic resection. (d) Pathological findings: well to moderately differentiated adenocarcinoma, pSM (3000–4000 *μ*m), ly(+), v(-), pN0. (B): (a) Conventional view: there is a small polyp (lesion size: 5 mm) located in the descending colon. It is difficult to visualize any depressed area in this lesion. (b) NBI view: magnification with NBI clearly demonstrates a depressed area in the center of this lesion. The vascular pattern in the lesion center is capillary pattern Type III_B_ by the Sano classification, suggesting the possibility of invasive cancer. Crystal violet staining is therefore required. (c) Crystal violet view: magnification with crystal violet staining demonstrates an invasive pattern. Invasive cancer cannot be predicted with high confidence because the depressed area is small. This lesion was treated by endoscopic mucosal resection for initial diagnosis, and later the patient underwent surgery. (d) Pathological findings: well differentiated adenocarcinoma with scirrhous growth, pSM massive, VM(+), ly(+), EMR.

Resection of all adenomatous polyps during colonoscopy has been the world standard treatment since the National Polyp Study demonstrated that colonoscopic resection of all adenomatous polyps reduced both the incidence of and mortality due to colorectal cancer [22, 23]. Up to now, all polyps have been routinely retrieved and submitted to pathology in view of the limited accuracy of conventional white light colonoscopy (59–84%) for differentiating neoplastic from nonneoplastic polyps [18, 34, 48–51]. Although magnifying chromoendoscopy using the pit pattern allows higher accuracy (85–96%) than conventional endoscopy or chromoendoscopy for differentiating polyps, the availability of both magnifying endoscopy and chromoendoscopy is unfortunately limited outside of Japan [18, 20, 50–52]. If it were possible to determine colorectal polyp pathology by endoscopy alone, recto-sigmoid hyperplastic polyps would be left *in situ* to reduce the risk of polypectomy, and small adenomas would be resected and then discarded to save the costs of histological evaluation. This concept, proposed in the UK and US, has been referred to as the “resect and discard” strategy, and has been limited to both diminutive (1–5 mm: US, UK) or small (6–9 mm: UK) polyps that appear histologically to have no malignant features and can be differentiated clinically with a high degree of confidence [21, 53]. Polyps for which diagnostic confidence is low are resected and sent for pathologic examination. The “resect and discard” strategy has considerable merits in terms of histology cost-saving and is thus expected to spread worldwide. However, discarding polyps without any histologic examination have an attendant risk of missing small invasive colorectal cancers that would normally be treated surgically. Kudo et al. reported that the frequency of small invasive cancers among all diminutive polyps (≤5 mm) was 0.16% (24/14892), and that macroscopically most of them were of the depressed type (22/24: 92%) [54]. Although such small invasive cancers are infrequent, they would be fatal if overlooked.  Figure 9 shows two cases of small invasive colorectal cancer. In order to find small invasive cancers, it is important to detect any depressed area present in a lesion.

Currently, many endoscopic modalities are available, including NBI, autofluence imaging, Fuji Intelligent Chromo Endoscopy, i-scan, and so on. What kind of modality can best determine colorectal polyp pathology endoscopically? The American Society for Gastrointestinal Endoscopy (ASGE) has suggested that the necessary thresholds of endoscopic technology for accurate assessment of histology are >90% agreement in determining postpolypectomy surveillance intervals and a negative predictive value of ≥90% (when used with high confidence) for recto-sigmoid polyps with adenomatous histology [55].  Table 4 demonstrates the performance of NBI without magnification for real-time assessment with high diagnostic confidence in the USA and UK [21, 53]. Both studies found that the accuracy was over 90%, but the the negative predictive value was 82% (<90%) in the UK and 95% in the USA. The lower negative predictive value in the UK study was explained by histological misclassification and loss or damage to polyps before histology. In both studies, the level of agreement in determining postpolypectomy surveillance intervals was over 90%. NBI might therefore become a useful modality for real-time assessment of the histology of diminutive polyps. Recently, Hewett et al. reported that use of NBI together with the NICE classification might also be useful, as the accuracy was 89% and the negative predictive value was 95% with high predictive value in real-time assessment [43]. However, more prospective research is needed to prove that this classification can be applied with satisfactory availability, feasibility, and reliability.

## 4. Summary

NBI with magnification colonoscopy is useful for histological prediction, and for estimating the depth of invasion of colorectal cancer. To standardize the diagnostic strategies currently available, the NICE classification would be helpful for endoscopists irrespective of whether they have access to magnifying endoscopy. However, more prospective research is needed to prove that this international classification can be applied with satisfactory availability, feasibility, and reliability. In the near future, NBI might make a valuable contribution to real-time histological prediction during colonoscopy, which would have substantial benefits for reducing both the risk of polypectomy and the costs of histological evaluation by allowing adenomatous polyps to be resected and discarded.

## Figures and Tables

**Table 1:** Comparison of endoscopic diagnosis of the depth of submucosal deeply invasive colon cancer.

Diagnostic method	Number of adenoma, m-ca^#^sm-slight-ca^##^	Number of sm deep-ca^###^	Overall accuracy (%)	Sensitivity (%)	Specificity (%)	PPV (%)	NPV (%)
Magnifying chromoendoscopy (Invasive pattern)	4035	180	98.8	85.6	99.4	86.5	99.4
NBI with magnifying colonoscopy (capillary pattern classification)	97	33	87.7	84.8	88.7	71.8	94.5
Nonlifting sign	245	26	94.8	61.5	98.4	80.0	96.0

^#^Intramucosal cancer.

^##^sm slight invasive (<1000 *μ*m) cancer.

^###^sm deep invasive (≥1000 *μ*m) cancer.

**Table 2:** NICE classification.

	Type 1	Type 2	Type 3
Color	Same or lighter than background	Browner relative to background (verify color arises from vessels)	Brown to dark brown relative to background; sometimes patchy whiter areas

Vessels	None, or isolated lacy vessels may be present coursing across the lesion	Brown vessels surrounding white structures**	Has area(s) of disrupted or missing vessels

Surface pattern	Dark or white spots of uniform size, or homogeneous absence of pattern	Oval, tubular or branched white structures** surrounded by brown vessels	Amorphous or absent surface pattern

Most likely pathology	Hyperplastic	Adenoma***	Deep submucosalinvasive cancer

Treatment	Followup	Polypectomy/EMR/ESD	Surgery

*Can be applied using colonoscopes with/without optical (zoom) magnification.

**These structures (regular or irregular) may represent the pits and the epithelium of the crypt opening.

***Type 2 consists of Vienna classification types 3, 4 and superficial 5 (all adenomas with either low or high grade dysplasia, or with superficial submucosal carcinoma). The presence of high grade dysplasia or superficial submucosal carcinoma may be suggested by an irregular vessel or surface pattern, and is often associated with atypical morphology (e.g., depressed area).

**Table 3:** NICE classification and Each NBI magnifying classification in Japan.

NICE clasification	Type1	Type2	Type3
Sano classification	Type I	Type II~IIIA	Type IIIB
Hiroshima classification	Type A	Type B~C2	Type C3
Showa classification	Faint pattern	Dens/Network pattern irregular pattern	Sparse pattern

**Table 4:** Performance of NBI without magnification for real-time assessment with high diagnostic confidence.

HC rate	Accuracy	Sensitivity	Specificity	PPV	NPV
78%	92%	93%	88%	93%	82%
80%	95%	91%	93%	91%	95%
